# Reduced Diversity and High Sponge Abundance on a Sedimented Indo-Pacific Reef System: Implications for Future Changes in Environmental Quality

**DOI:** 10.1371/journal.pone.0085253

**Published:** 2014-01-24

**Authors:** Abigail Powell, David J. Smith, Leanne J. Hepburn, Timothy Jones, Jade Berman, Jamaluddin Jompa, James J. Bell

**Affiliations:** 1 School of Biological Sciences, Victoria University of Wellington, Wellington, New Zealand; 2 Coral Reef Research Unit, University of Essex, Colchester, United Kingdom; 3 Research and Development Center on Marine, Coastal and Small Islands, Hasanuddin University, Makassar, Indonesia; University of Genova, Italy

## Abstract

Although coral reef health across the globe is declining as a result of anthropogenic impacts, relatively little is known of how environmental variability influences reef organisms other than corals and fish. Sponges are an important component of coral reef fauna that perform many important functional roles and changes in their abundance and diversity as a result of environmental change has the potential to affect overall reef ecosystem functioning. In this study, we examined patterns of sponge biodiversity and abundance across a range of environments to assess the potential key drivers of differences in benthic community structure. We found that sponge assemblages were significantly different across the study sites, but were dominated by one species *Lamellodysidea herbacea* (42% of all sponges patches recorded) and that the differential rate of sediment deposition was the most important variable driving differences in abundance patterns. *Lamellodysidea herbacea* abundance was positively associated with sedimentation rates, while total sponge abundance excluding *Lamellodysidea herbacea* was negatively associated with rates of sedimentation. Overall variation in sponge assemblage composition was correlated with a number of variables although each variable explained only a small amount of the overall variation. Although sponge abundance remained similar across environments, diversity was negatively affected by sedimentation, with the most sedimented sites being dominated by a single sponge species. Our study shows how some sponge species are able to tolerate high levels of sediment and that any transition of coral reefs to more sedimented states may result in a shift to a low diversity sponge dominated system, which is likely to have subsequent effects on ecosystem functioning.

## Introduction

The plight of tropical coral reefs has been well reported with an estimated 60% of reefs being threatened by local-scale human activities, including coastal development, overexploitation, invasive species and pollution [1]. In addition, all coral reefs across the world face the threat of global warming and ocean acidification [2]. The Indo-Pacific region is a global hotspot of marine diversity for most major taxa [3] and encompasses 75% of the world's coral reefs; however, this region has also experienced widespread degradation of its reefs. An analysis of 2667 surveys of Indo-Pacific coral reefs between 1968 and 2004 suggest that an average of 1% or 1,500 km^2^ of hard coral cover has been lost per year over this period [4]. Despite high research effort, high public interest and the known societal importance of Indo-Pacific reefs, there is a paucity of information concerning the ecology of reef-associated fauna other than reef building corals and fish. Consequently, there is an urgent need for greater research effort focusing on the effects of environmental degradation on those fauna that play pivotal roles in driving the community structure of tropical reefs [5].

After corals, sponges are one of the most dominant benthic taxa inhabiting coral reefs [6] and play many important functional roles, particularly through their interactions with the water column, biogeochemical cycling, nutrient cycling, bioerosion, and their facilitation of primary production as a result of their symbiotic associations with microbes [7]–[9]. In addition, due to their competitive ability and well-documented interactions with other reef organisms [10], changes in the distribution, composition and abundance patterns of sponges are likely to affect overall reef ecosystem functioning (e.g. nutrient recycling and spatial interactions).

So called “phase shifts” have been widely associated with declining reef quality and reduced herbivore abundance [11] and most often reefs have changed from coral to algal-dominated states. However, there also appears to be the potential for coral-dominated systems to change to states dominated by sponges [12], [13]. For example, increases in the bioeroding sponge *Cliona caribbaea* were observed on coral reefs in Puerto Rico following declines in hard coral cover resulting from the combined effects of disease, hurricane damage, siltation and eutrophication [14], while reefs in Belize experienced large increases in the sponge *Chondrilla nucula* following a thermal anomaly driving mass coral mortality [15]. In fact, in the Caribbean sponge biomass has now been suggested to exceed live coral biomass [6], and while coral declines have occurred in the past [16], the abundance of some important sponge species have increased [17]. Although such changes to sponge-dominated states have mainly been reported from the Caribbean and for boring sponge species, there is also some evidence they are more widespread and including non-boring forms [18], [19]. Furthermore, there is increasing evidence that sponges may benefit from any future declines in coral as a result of ocean acidification or ocean warming [13].

In order to successfully ascertain the possibility of reefs to experience changes to sponge-dominated states, and to fully understand the impact of future climate change and ocean acidification, it is important to identify and evaluate the relative importance of those environmental variables that influence the abundance, distribution and composition of sponge assemblages. For example, determining the extent to which the abundance of sponges is influenced by spongivorous fish may give us some insight into the potential cascade effects of the overexploitation and removal of such fish from the system. Alternatively, if abiotic variables, such as sedimentation, are important drivers of abundance and assemblage structure, then sponge assemblages may be more affected by processes such as coastal development or mangrove clearing [20], [21]. Furthermore, understanding sponge driven ecological interactions at the community level will help us determine long-term consequences of anthropogenic stressors that influence sponge abundance and biodiversity. Many studies have found correlations between one or two abiotic variables and sponge abundance and diversity [22]. However, few studies have considered multiple potential drivers in a single study; this inhibits our ability to identify the most important variables driving patterns of abundance and diversity within a given system (but see [18], [23]).

The most important abiotic variables influencing sponge diversity, abundance and distribution patterns include: sedimentation [23]–[25]; water flow [24], [26], [27]; wave action [28]–[30]; substrate type [31], [32]; and light [33]. The most important biotic variables influencing sponge distribution and abundance patterns include: predation (from fish, turtles and starfish) [34]–[38]; spatial competition [27], [39]–[42]; and food availability [43]. However, the perceived importance of biological variables in influencing sponge abundance, diversity and distribution patterns is primarily from research conducted in the Caribbean, and their importance for wider coral reef sponges is unknown.

Given the global concern about declining reef quality, we studied the sponge assemblages on an Indonesian coral reef system at sites with different biological and physical characteristics. The aim of our study was to identify the biological and physical variables explaining differences in sponge abundance, diversity and assemblage composition among locations and to determine the potential for sponges to become more abundant in response to changing environmental conditions.

## Materials and Methods

### Study sites

**Figure 1:**
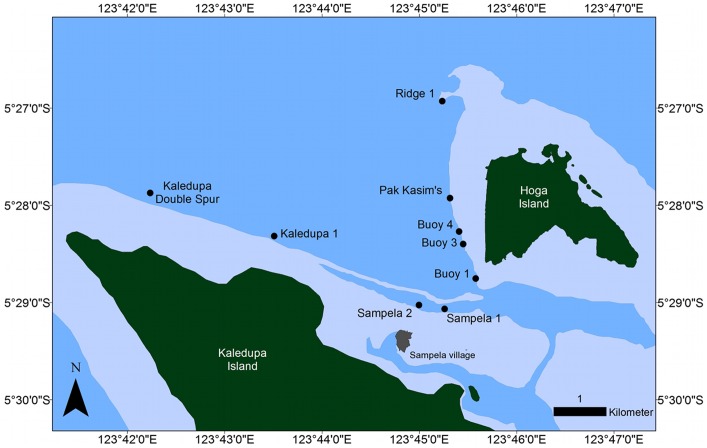
Map of study sites. Location of the nine study sites in the Wakatobi Marine National Park.

This study was conducted in the Wakatobi Marine National Park (WMNP) in southeast Sulawesi in Indonesia. Research Permits were issued by the Indonesian State Ministry for Research and Technology (RISTEK) and the Indonesian Institute of Sciences (PPO-LIPI). The WMNP was gazetted in 1996 and is the third largest marine national park in Indonesia [44]. The park is located in the coral triangle and supports highly diverse marine communities, but is also inhabited by over 90,000 people who are heavily dependent on reef resources for food and income [45]. Declines in hard coral cover have been documented in the park since 2002 [46] and there is also evidence that a number of nearshore fisheries are currently being exploited at unsustainable levels [47]. Environmental and biological variables were quantified on upper reef slopes at nine sites (Fig. 1) at a depth of approximately 10 (±1) m. These sites were selected as they encompass a range of environmental and biological conditions particularly with respect to benthic assemblage composition, coral cover, fish assemblages and sedimentation levels. Surveys were carried out between 07:00 and 17:00 from June-August 2010.

Five sites, locally known as Pak Kasim's, Buoy 1, Buoy 3, Buoy 4 and Ridge 1 are located on the fringing reef on the western side of Hoga Island. Buoy 1 is situated at the south western tip of Hoga Island in the channel between Hoga and Kaledupa Island. The reef slope drops off steeply (>70°) and is characterised by caves and overhangs, turning into a sandy slope at approximately 40 m. Buoy 3 and Buoy 4 are predominately vertical wall sites (>70°) with some overhangs. Pak Kasim's is located approximately 500 m north of Buoy 4 and at this site the reef slope aspect is less steep (50–70°). The last site, Ridge 1, runs north-south approximately 700 m offshore from Hoga. The top of the ridge is located at 1–2 m and descends steeply (60–80°) dropping to over 100 m. Surveys were carried out on the western side of the Ridge.

Four further sites, Kaledupa Double Spur, Kaledupa, Sampela 2 and Sampela 1 are located on the northern side of Kaledupa on the fringing reef that surrounds the island. Kaledupa Double Spur is located at the northern tip of Kaledupa Island. Surveys at this site were carried out on a vertical reef wall characterised by large gorgonians and sponges that descends to over 70 m. At the Kaledupa site, the reef crest is located 300 m offshore adjacent to seagrass beds and areas of mangrove forest. The reef slope has a more gentle aspect than Kaledupa Double Spur, descending at an angle of approximately 50° to depths of over 50 m. Sampela 1 is adjacent to a Bajo village of approximately 2000 people constructed on the reef flat and is subject to high levels of anthropogenic disturbance including invertebrate and fish extraction, coral mining and likely experiences high levels of nutrient and suspended solid input. The reef slope descends at a 45° angle and is characterised by low hard coral cover (3%) and high sedimentation rates [48]. Sampela 2 is located approximately 250 m further north from Sampela 1 and both sites have similar site characteristics. However, earlier surveys indicate that Sampela 1 has slightly higher levels of hard coral cover than Sampela 2 and is less impacted by sedimentation [49].

### Biological variables

The abundance and diversity of sponges were surveyed *in situ* using SCUBA in six 1 m^2^ divided quadrats at each site (total n = 54). To identify a suitable sample size for the replication within each site, species accumulation curves based on surveys of 15 permanent monitoring quadrats (Bell unpublished data) were examined. Accumulation curves were produced using the statistical software package PRIMER for the observed number of species given the sample sizes ranging from 1–15 (figure S1). Six quadrats were surveyed at each site as this captured an average of 80% (46 of 58 species, with a 95% CI of 38 to 54 species) of the species captured in 15 quadrats and further quadrats provide limited returns in terms of the new species detected (8 quadrats: 50 species, 10 quadrats: 54 species), whilst significantly increasing the time spent performing the surveys. Quadrats were placed at random pre-selected positions along a 30 m transect tape, which was laid out following a 10 m depth contour on the reef slope. The location of the quadrats was determined using a random number generator (six random positions selected from 0–29 for each transect) prior to the surveys. If the pre-selected quadrat location happened to be an unsuitable location, such as a cave or overhang, the quadrat was moved to the nearest suitable reef slope/wall habitat location further along the tape. Sponge patches were counted if any part of the sponge was in the quadrat. We chose to measure the number of sponge patches rather than biomass or area occupied because: 1) the nature of the sponge assemblages in the region, which are dominated by small encrusting patches see [50]; 2) earlier studies in the region have shown a strong correlation between area occupied and number of patches, and similar multivariate assemblage patterns see [25]; 3) the topographic complexity of the study sites meaning it was not possible to estimate area occupied from photographs. All sponge species were assigned an ID code, which were considered Operational Taxonomic Units (OTUs) for the purposes of this study. To confirm the consistency and validity of these units, photographs were taken of each OTU, along with small samples that were used for spicule preparations and sections [51]. Habitat quality was defined by the percentage cover of hard coral, coralline algae, other algae (turf or macro), soft coral, rock, rubble, sand, and other (e.g. ascidians, bryozoans) calculated from photo-quadrats (n points = 100) using Coral Point Count [52]. Fish surveys were carried out between 10:00–15:00 along three 50×5 m transects on the reef slope (10 m) at each site using the time (15 mins) and distance restricted underwater visual census method see [53]. Spongivorous fish were classified as: a) those reported in the literature as having a diet of >5% sponge (from gut contents) [54]–[59] b) from a preliminary analysis of fish gut contents carried out as part of this study; and c) species observed feeding on sponges during this survey.

### Environmental variables

Turbidity, temperature and water column chlorophyll-a (as a proxy for food availability) were recorded using an RBR XR-420 data logger set to record every minute with no averaging. Water flow was measured with an impeller current meter (Valeport Model 106). The XR-420 data logger and flowmeter were deployed on reef slopes at a depth of 10 m for a minimum of three 24 hour periods at each study site. To quantify sedimentation levels, four sediment traps were deployed on the reef slope at each site (10 m) approximately 5 m apart. Sediment traps were constructed as described in [53]. After 10 days the traps were sealed and collected. The sediment was collected using filter paper and dried in an oven at 100°C for a minimum of 24 hours. The sediment samples were then weighed to obtain mean dry weight of sediment at each site. The angle of the reef surface within each quadrat was measured using a pivoted protractor mounted on a spirit level.

### Statistical analyses

Statistical analyses were performed in the PRIMER-E v6 environment (Plymouth Routines In Multivariate Ecological Research) with the PERMANOVA add-on. Analyses were based on resemblance matrices calculated using Bray-Curtis similarity coefficients.

### Benthic characteristics of the study sites

A one-factor permutational multivariate analysis of variance (PERMANOVA) was used to test for differences in the benthic characteristics of the study sites with site as a fixed factor with nine levels. PERMANOVA was used as it is a permutation-based method and therefore makes no assumption about the distribution of the data. Site differences were represented graphically using unconstrained non-metric Multi Dimensional Scaling (MDS) and constrained Canonical Analysis of Principal Coordinates (CAP). Spearman rank correlations between individual benthic components and the resulting CAP axes were used to identify benthic groups that were characteristic of particular study sites.

### Sponge abundance patterns

Differences in total sponge abundance among study sites was tested using a one factor PERMANOVA (PERMANOVA operating on univariate data is equivalent to a permutational ANOVA) with site as a fixed factor with nine levels. The associations between sponge abundance and biotic/environmental variables were investigated using distance-based multiple linear regression (DISTLM). DISTLM is a routine that can be used to model the relationship between a multivariate distance based dataset (whether the distances are calculated from multivariate assemblage data or between univariate data points), as described by a resemblance matrix, and a set of predictor variables [60]. Draftsman plots were used to check for skewness and multi-collinearity in the predictor variables. Variables that were highly correlated with other variables were removed in order to maximise the parsimony of our models. Turbidity was excluded from the analysis as it was highly correlated (R^2^>0.9) with sediment levels. The following 10 variables were considered in the DISTLM analysis: substrate angle, temperature, sediment, flow rate, chlorophyll-a, hard coral cover, coralline algae, other non-coralline algae, soft coral and spongivorous fish abundance. Models incorporating all possible combinations of predictor variables were generated using the *Best* procedure within the DISTLM routine. We then used an information theoretic approach based on modified Akaike's Information Criterion (AICc) to identify the best model. AICc values indicate the goodness of a model fit to the data, penalised for increasing the number of variables [61]. Models with the lowest AICc are considered the most parsimonious. We discuss the model with the lowest AICc value, but we also calculated the Akaike weights of all models with AICc values within five of the best model. The Akaike weight, *w_i_*, for a given model, denoted by subscript *i*, is calculated as
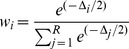
where R is the number of candidate models and Δ*_i_* is the difference in AICc value between model *i* and the model with the lowest AICc value. The Akaike weight demonstrates the best model relative to others [62]. Assessing the Akaike weights therefore allows us to identify and quantify the uncertainty in model selection. The Akaike weights were also used to estimate the relative importance of each predictor variable [61]. For each predictor, the Akaike weights of all the models (with ΔAICc less than 5) that contained that predictor were summed. The summed Akaike weights for each predictor can be interpreted as the relative importance of that predictor. Those predictors that consistently occur in the most likely models have an Akaike weight close to 1 whereas variables that are absent from or are only present in poorly fitting models (high AICc values) have an Akaike weight close to 0 [61].

### Sponge diversity and assemblage patterns

Species diversity indices were calculated for each study site based on the OTUs. We measured the total number of species present (S), Shannon's index (H') and Pielou's evenness index (J'). Species rank abundance curves were also generated to examine sponge assemblage structure at the different study sites. To examine the relationship between local abundance (within each site) and prevalence (among sites) we identified the maximum abundance and prevalence of each species [63]. The abundances for each species were summed across quadrats within each site and the maximum of these summed site specific values (standardised to abundance per m^2^) was taken as a measure of its local abundance. These values were then plotted against its prevalence among sites ( = number of occupied sites/9). A log transformation was applied to local abundance to control for extremely high values and to achieve a relatively equal spread. A simple linear regression (normal errors) was applied to the data to further examine the relationship between abundance and occupancy and R^2^ and pearson correlation coefficient values were calculated. These analyses were performed in R version 3.0.2 [64].

The same PERMANOVA design was used as described above to test for differences in the multivariate sponge assemblages at the study sites. Prior to analysis a dispersion weighting transformation was applied to the data to down-weigh the importance of numerically abundant species. This transformation was considered appropriate as some sponges, particularly *Lamellodysidea herbacea*, were highly abundant and also showed evidence of spatial clustering [65]. Constrained analysis of principal coordinates (CAP) was used to visualise the differences in the sponge assemblages and to identify species that were characteristic of the different study sites. Spearman rank correlations (>0.4) of individual species abundances with the CAP axes were used to determine which species were most characteristic of the study sites. The associations between sponge assemblage structure and the other variables were investigated using the same approach as our analysis of sponge abundance using permutational distance-based multivariate multiple regression (DISTLM).

## Results

### Environmental variables and benthic characteristic of the study sites

**Figure 2:**
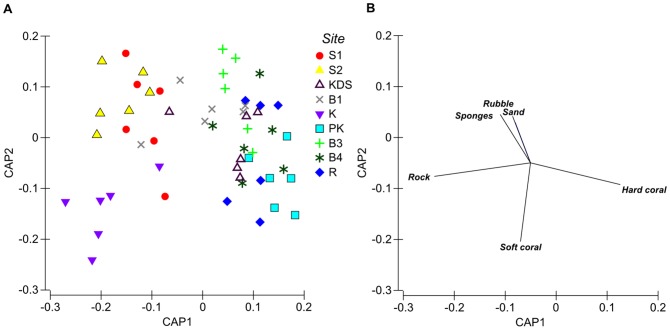
Benthic assemblage composition at the study sites. Canonical Analysis of Principal Coordinates (CAP) plot showing differences in benthic composition between study sites. Vectors are overlaid for benthic components that have a Spearman rank correlation greater than 0.4 with either of the resulting CAP axes.

The environmental and biological parameters measured at each site are summarised in Table 1. Mean quadrat angle ranged from 78±5.66° (±1 SD) at Buoy 4 to 46.67±31.09° at Sampela 1. Chlorophyll-*a* also varied between sites with the highest mean value recorded at Kaledupa (0.42±0.19 µg/l) and the lowest at Pak Kasim's (0.14±0.06 µg/l). There was little variation in the mean water temperatures recorded on the reef slopes at the study sites. Temperatures ranged from 28.12±0.18°C at Kaledupa to 27.37±0.69°C at Buoy 1. The highest mean flow rate was recorded at Sampela 1 (0.063±0.044 m/s) and the lowest was recorded at Buoy 1 (0.002±0.0084 m/s). Mean spongivore fish abundance varied between 19±4.16 per 250 m^2^ transect at Buoy 3 to 48±11.36 per 250 m^2^ at Sampela 1. Benthic assemblage composition was significantly different between study sites (PERMANOVA, Pseudo-F = 5.6497, p = 0.0001). Overall allocation success from the CAP analysis was 40.74% (Fig. 2). The results of the CAP analysis of benthic characteristics (Fig. 2) showed that: Sampela 1 and Sampela 2 were characterized by sponges, sand and rubble; Kaledupa 1 was characterised by rock and soft corals; and Ridge 1, Pak Kasim's, Buoy 1, Buoy 3, Buoy 4 and Kaledupa Double Spur were all characterized by hard coral.

**Table 1:** Summary table of environmental and biological predictor variables.

Variable	Units	Site								
		Sampela 1	Sampela 2	Kaledupa	Kaledupa Double Spur	Buoy 1	Buoy 3	Buoy 4	Pak Kasim's	Ridge 1
Angle per site	°	46.67 (±31.09)	50 (±16)	52.67 (±20.7)	62.83 (±33.86)	58.33 (±25.43)	64.17 (±14.63)	78 (±5.66)	66.67 (±19.92)	65 (±21.68)
Temperature	°C	27.73 (±0.12)	27.67 (±0.32)	28.12 (±0.18)	27.92 (±0.21)	27.37 (±0.13)	27.91 (±0.04)	27.82 (±0.37)	28.06 (±0.13)	27.66 (±0.13)
Turbidity	Standard turbidity units	3.88 (±4.58)	2.92 (±0.98)	0.26 (±0.17)	0.78 (±0.38)	1.45 (±0.69)	0.72 (±0.74)	0.76 (±0.63)	0.93 (±1.06)	0.19 (±0.33)
Chlorophyll-*a*	µg/l	0.39 (±0.1)	0.39 (±0.12)	0.42 (±0.19)	0.26 (±0.08)	0.25 (±0.13)	0.33 (±0.08)	0.26 (±0.14)	0.14 (±0.06)	0.33 (±0.08)
Sediment	g dry weight /day	0.357 (±0.168)	0.322 (±0.051)	0.124 (±0.019)	0.109 (±0.047)	0.162 (±0.029)	0.235 (±0.202)	0.168 (±0.099)	0.122 (±0.065)	0.112 (±0.022)
Flow	m/s	0.063 (±0.044)	0.056 (±0.038)	0.014 (±0.02)	0.038 (±0.041)	0.0022 (±0.0084)	0.02 (±0.024)	0.018 (±0.023)	0.028 (±0.033)	0.04 (±0.046)
Hard coral	% Cover	11.11 (±7)	7.73 (±5.43)	4.63 (±3.77)	27.49 (±14.86)	23.36 (±11.26)	31.45 (±16.74)	45.59 (±15.18)	57.05 (±13.83)	35.7 (±13.62)
Soft coral	% Cover	6.39 (±5.44)	2.32 (±2.7)	22.13 (±10.15)	13.08 (±8.39)	4.96 (±3.03)	1.38 (±1.79)	3.88 (±5.76)	11.11 (±3.9)	17.34 (±16.96)
Coralline algae	% Cover	14.38 (±15.53)	14.12 (±7.79)	12.15 (±6.79)	27.43 (±11.75)	20.81 (±11.21)	27.34 (±24)	22.32 (±15.82)	15.12 (±6.72)	12.23 (±10.66)
Other non-coralline algae	% Cover	6.8 (±6.8)	1.12 (±4.83)	2.73 (±1.8)	2.97 (±2.44)	4.18 (±2.44)	4.16 (±2.44)	2.37 (±2.44)	2.27 (±1.75)	6.86 (±5.02)
Spongivorous fish	Number/125 m^2^	48 (±11.36)	37 (±8.33)	28 (±9.64)	37 (±8.08)	26 (±1.15)	19 (±4.16)	24 (±6.11)	23 (±7.81)	30 (±7.81)

### Sponge abundance patterns

**Figure 3:**
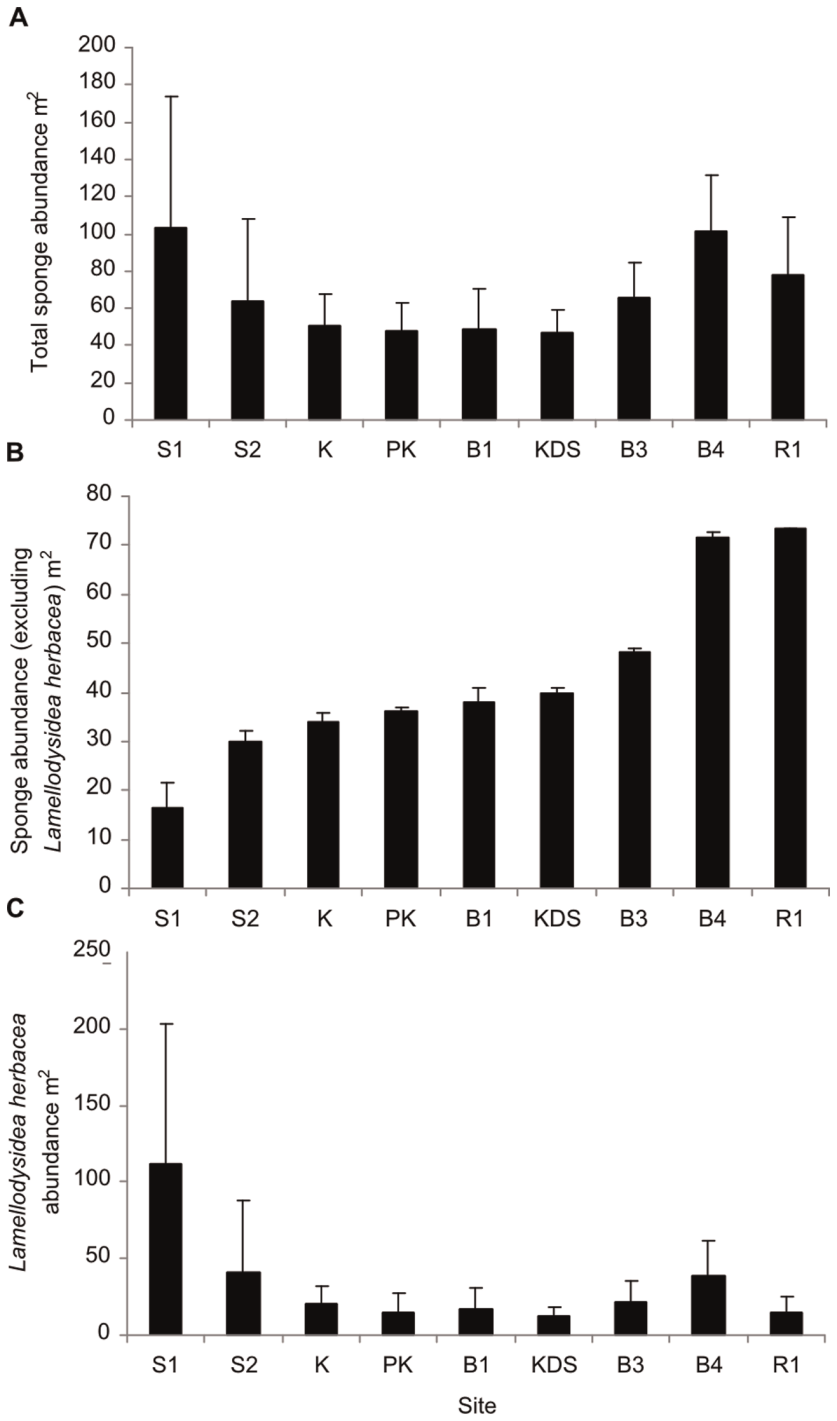
Mean sponge abundance. a) Mean sponge abundance at the nine study sites. b) Mean sponge abundance excluding *Lamellodysidea herbacea* at the study sites. c) Mean *Lamellodysidea herbacea* abundance at the study sites.

**Figure 4:**
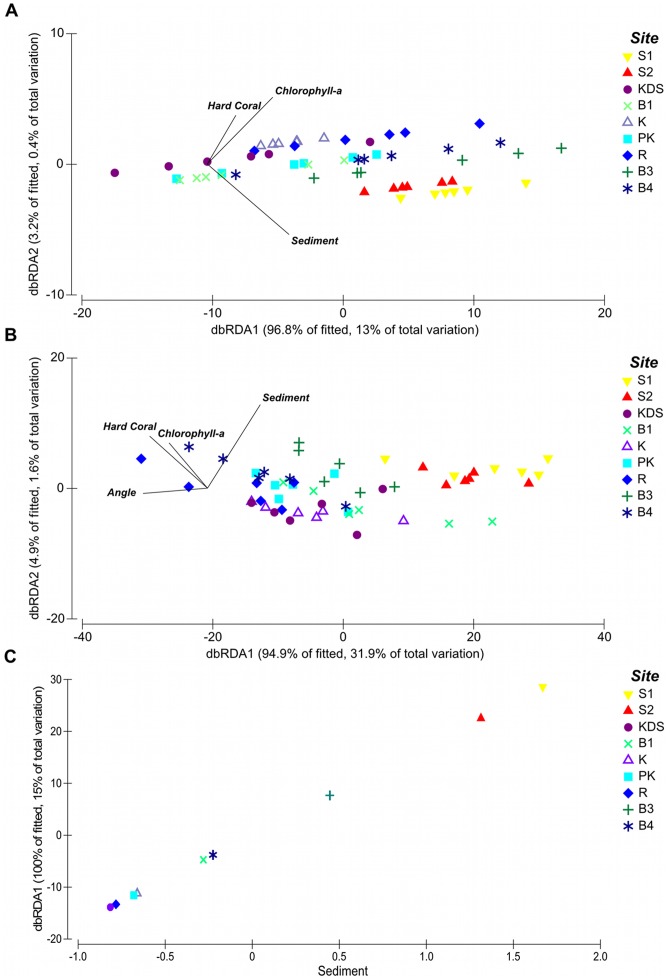
Distance based redundancy analysis (DbRDA) ordinations of fitted models. Distance based redundancy analysis (DbRDA) ordinations of models with the lowest AICc value of all competing models for: a) total sponge abundance; b) sponge abundance when *Lamellodysidea herbacea* was excluded; and c) *Lamellodysidea herbacea* abundance.

**Figure 5:**
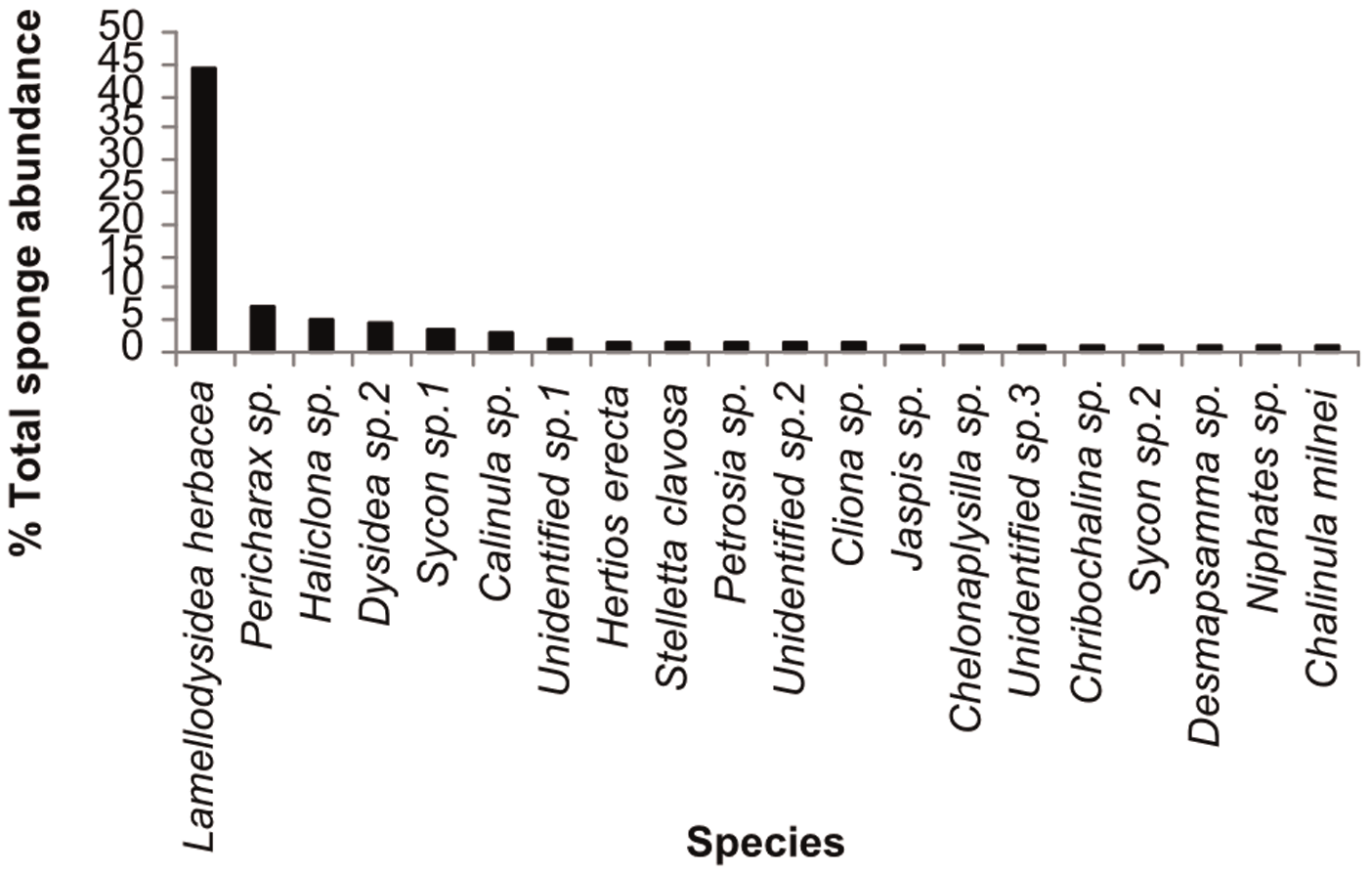
Sponge species relative abundance. Dominance plot showing the percentage abundance of the 20 most abundant sponge species.

In total, we counted 3856 sponges across all the study sites with a mean density of 71 (±78) sponges per m^2^. Total sponge abundance was significantly different between study sites (PERMANOVA, Pseudo-F = 2.6566, p = 0.0073). Sponge abundance was highest at Sampela 1 (103.67±69.68 per m^2^) and lowest at Kaledupa Double Spur (47±11.93 per m^2^), although there were considerable differences in the levels of variation between the quadrats at the different sites (Fig. 3). The results of the DISTLM analysis for total sponge abundance showed that the best model for sponge abundance contained three predictor variables, but only explained a small amount of the total observed variation (13%) (see Fig. 4 and Table 2); hard coral cover (8%), sediment (4%) and chlorophyll-*a* (1%). However, further analysis of the abundance data showed that one species *Lamellodysidea herbacea* accounted for 42% of all sponges observed across all sites (1613 sponges in total; Fig. 5). Therefore, we re-analysed our data to examine: 1) the variables that were associated with *Lamellodysidea herbacea* abundance; and 2) the variables associated with the abundance of the rest of the sponge assemblages.

**Table 2:** Summary table of the results of the DISTLM analysis, results shown are for the model with the lowest AICc values for each response variable.

Response	AICc	% total variability explained	Predictors	% variability explained by each predictor	Pseudo-F	P-value	Relationship
Total sponge abundance	333.08	13	Hard coral cover	8	0.47	0.57	positive
			Sediment	4	2.67	0.092	positive
			Chlorophyll-a	1	0.96	0.34	positive
Sponge abundance excluding	340.71	34	Sediment	18	12.1	0.0004	negative
*L. herbacea*			Quadrat Angle	12	7.64	0.0029	positive
			Hard coral cover	1	6.58	0.0064	positive
			Chlorophyll-a	4	2.38	0.12	unclear
Abundance of *L. herbacea*	390.85	15	Sediment	15	9.2	0.0001	positive
Multivariate sponge assemblage	426.74	26	Sediment	8	5.06	0.0001	NA
			Chlorophyll-a	6	3.64	0.0001	NA
			Spongivorous fish	6	3.45	0.0001	NA
			Flow	5	2.84	0.0001	NA
			Temperature	4	2.2014	0.001	NA

When we modeled sponge abundance excluding *Lamellodysidea herbacea*, the best model (based on AICc) contained four predictor variables that together explained 34% of the variation in sponge abundance between quadrats (Fig. 4 and Table 2); sedimentation (18% of total variation explained), quadrat angle (12%), chlorophyll-*a* (4%) and hard coral cover (1%) (see Table 3). We found that mean sponge abundance was negatively correlated with mean sedimentation at the study sites (Table 3), with the highest sponge abundance (excluding *Lamellodysidea herbacea*) at sites with the lowest sedimentation, such as Ridge 1 and Buoy 4, and lowest at the highly sedimented sites Sampela 1 and Sampela 2. The results of the summed Akaike weights for each parameter ranged from 0.92 to 0.16 (see table 3). Sedimentation had a summed Akaike weight of 0.92 indicating that it was found in the vast majority of the most likely models. Quadrat angle also had a high weight (0.73). Hard coral cover and chlorophyll-*a* had Akaike weights of 0.62 and 0.53, respectively, indicating lower support for either of these variables than for sedimentation or quadrat angle. Spongivorous fish was not included in the best model, but had a summed Akaike weight across all models of 0.61; this indicates that although it wasn't in the best overall model there is some support that this variable is correlated with sponge abundance. All of the other parameters had Akaike weights of less than 0.5 indicating little support for their association with sponge abundance (Table 3).

**Table 3:** Table showing the summed Akaike weights for each parameter for all models within Δ AICc of five for each of the response variables.

	Summed parameter Aikaike weights for each response variable			
Predictors	Total sponge abundance	Sponge abundance excluding *L. herbacea*	Abundance of *L. herbacea*	Multivariate sponge assemblage
Sediment	0.47	0.92	0.98	0.72
Spongivorous fish	0.2	0.61	0.31	0.53
Chlorophyll-*a*	0.53	0.53	0.22	0.52
Temperature	0.2	0.42	0.23	0.52
Flow	0.26	0.35	0.25	0.49
Hard coral	0.65	0.62	0.19	0.44
Quadrat angle	0.19	0.73	0.21	0.38
Coralline algae	0.28	0.29	0.42	0.37
Soft coral	0.19	0.23	0.29	0.34
Other algae	0.27	0.16	0.29	0.23

The DISTLM analysis for *Lamellodysidea herbacea* showed that the best model contained only one variable, sediment, explaining 15% of the variation in *L. herbacea* abundance between quadrats. In contrast to the abundance of sponges excluding *L. herbacea*, the abundance of *L. herbacea* was positively correlated with sedimentation rates (Table 2). This species was most abundant at the sites with the lowest habitat quality, Sampela 1 and Sampela 2. The abundance of *L. herbacea* was also highly variable between quadrats as they tended to be found in large aggregations in some quadrats but were absent from others. The results of the summed Akaike weights for each parameter ranged from 0.98 to 0.19 (see Table 3); however, sedimentation had a much higher summed Akaike weight than any other variable (0.98) and was therefore the most important variable explaining the abundance patterns of this species.

### Sponge diversity and assemblage patterns

**Figure 6:**
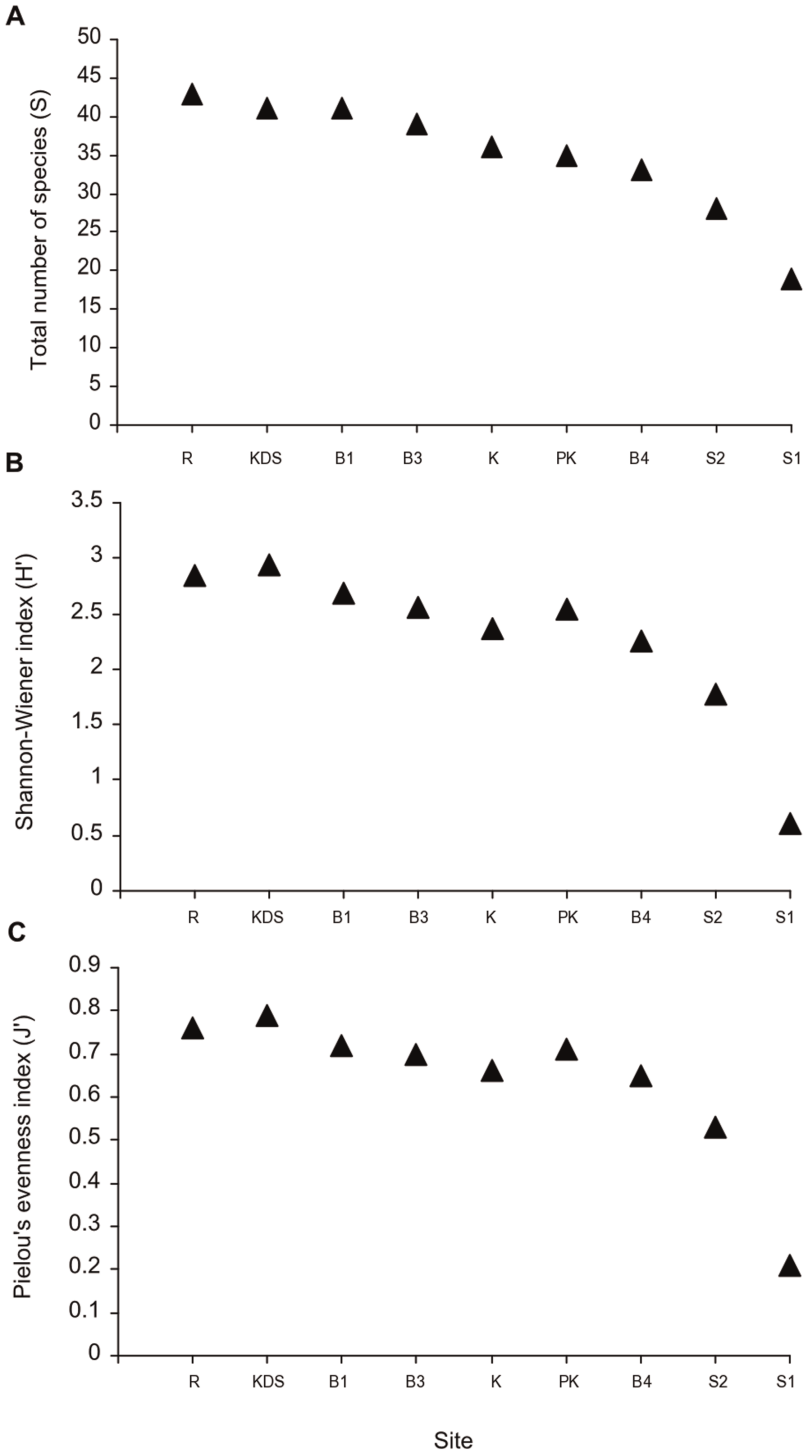
Sponge species diversity. Species diversity measures: a) total number of species; b) Shannon-Wiener index (H') c) Pielou's evenness index (J').

**Figure 7:**
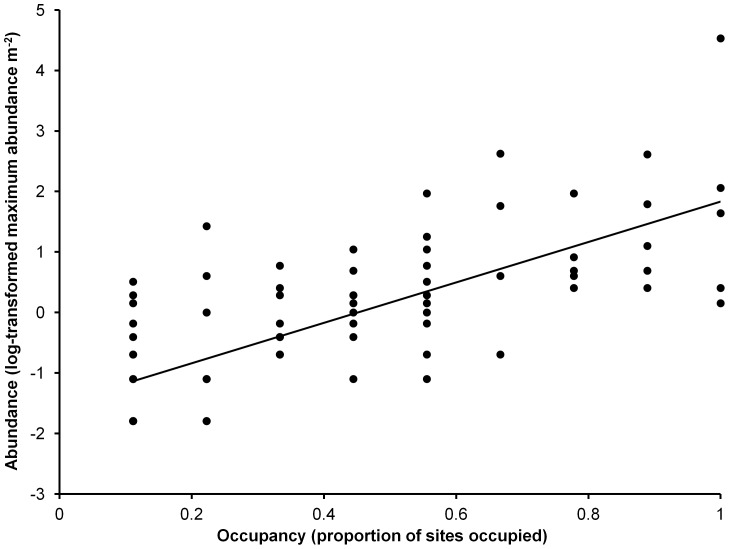
Occupancy-frequency distribution. Relationship between log abundance (maximum of the site-specific abundances) and occupancy. Each data point represents a different species (n = 85).

The three sites with the highest total species richness were Ridge 1 (S = 43), Buoy 1 (S = 41) and Kaledupa Double Spur (S = 40) (Fig. 6). These sites also had the highest Shannon diversity and Pielou's evenness. The sites with the lowest species richness were Sampela 1 (S = 19) and Sampela 2 (S = 28); these sites also had the lowest species diversity and evenness. Pielou's evenness index showed that Sampela 1, and to a lesser extent Sampela 2, were dominated by only a few species compared to the higher quality sites. This was also evident in the species abundance curves (figure S2). The sponge assemblages at Sampela 1 and to a lesser extent Sampela 2 were characterized by a skewed pattern with high abundances of one species. In contrast sponge assemblages at the other sites were more even with abundances more evenly distributed across more species. Plotting local abundance against prevalence revealed that the species that tended to be locally abundant were also widespread, whilst locally scarce species also tended to be present at fewer sites (Fig. 7). The relationship between local abundance and prevalence was highly significant (p-value<0.001) with a Pearson correlation coefficient of 0.73 and an R^2^ value of 0.53 from the linear model fit.

**Figure 8:**
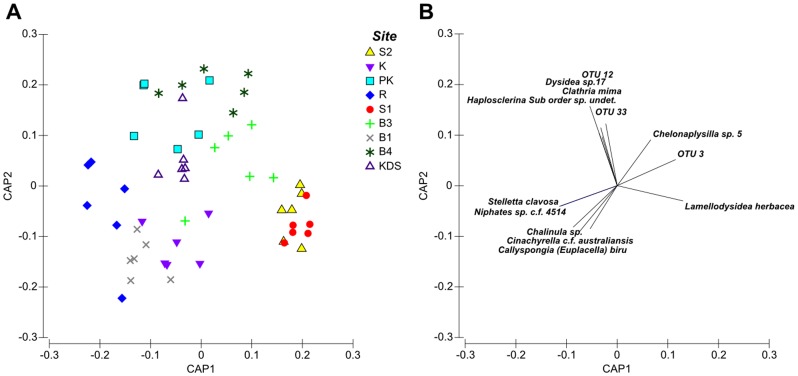
Sponge assemblage composition at the study sites. Canonical Analysis of Principal Coordinates (CAP) plot showing the differences in sponge assemblages between the study sites. Vectors indicate species that were characteristic of the overall sponge assemblage at each of the sites.

PERMANOVA tests revealed that there was a significant difference between the sponge assemblages at the study sites (pseudo-F = 3.12, p<0.001). Sponge assemblages at Sampela 1 and Sampela 2 were similar, and characterised by high abundance of *Lamellodysidea herbacea* (Fig. 8). Sponge assemblages at Kaledupa, Buoy 1 and Ridge 1 were characterised by *Stelletta clavosa*, *Callyspongia (Euplacella) biru* and *Cinachyrella c.f. australiensis* Pak Kasim's and Kaledupa Double Spur, were characterised by *Clathria (Microciona) mim*a, *Dysidea* sp. 12, and *Haplosclerina* sp. Finally, Buoy 3 and Buoy 4 were characterised by *Chelonaplysilla* sp 5.

**Figure 9:**
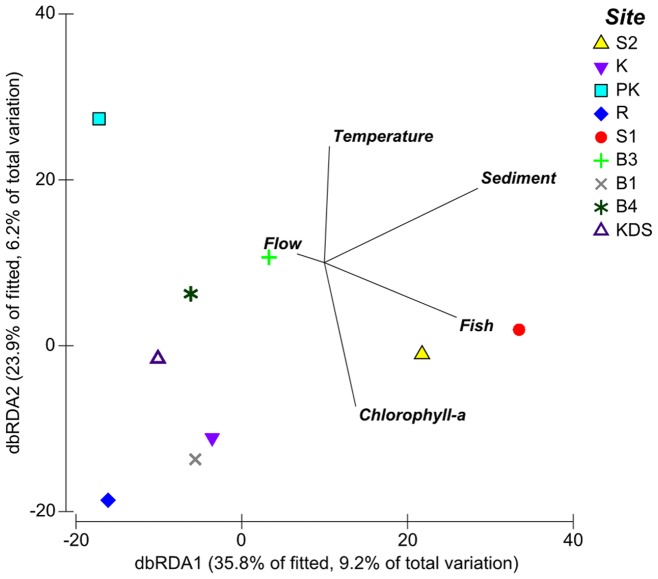
Factors associated with observed variation in sponge assemblages at the study sites. Distance based redundancy ordination (DbRDA) of the model with the lowest AICc value for the multivariate sponge assemblage data.

The best model identified using DISTLM explained 26% of the variation in sponge assemblages between quadrats and contained five variables: sediment (8%), chlorophyll-a (6%), spongivorous fish abundance (6%), flow rate (5%), and temperature (4%) (Fig. 9). The Akaike weights of all the predictor variables ranged from 0.72 to 0.23 (Table 4). Spongivorous fish abundance, chlorophyll-a and temperature had Akaike weights greater than 0.5 providing some support for these variables being correlated with sponge assemblage structure, but the major contributor was sediment with an Akaike weight of 0.72.

**Table 4:** List of spongivorous fish and the percentage sponge found in gut contents.

Family	Species	% Gut Contents	Location	Reference
Acanthuridae	*Ctenochaetus binotatus*		Sulawesi, Indonesia	Personal observation of feeding behavior
Acanthuridae	*Acanthurus pyroferus*		Sulawesi, Indonesia	Personal observation of feeding behavior
Balistidae	*Sufflamen bursa*	5.88	Hawaii, USA	Hobson, E. S., 1974
Blenidae	*Ecsenius pictus*		Sulawesi, Indonesia	Personal observation of feeding behavior
Chaetodontidae	*Chaetodon bennetti*	13	Kalimantan, Indonesia	Nagelkerken et al 2009
Chaetodontidae	*Chaetodon kleinii*	8	Kalimantan, Indonesia	Nagelkerken et al 2009
Chaetodontidae	*Chaetodon lunulatus*	5	Kalimantan, Indonesia	Nagelkerken et al 2009
Chaetodontidae	*Chaetodon unimaculatus*	17.49	Hawaii, USA	Hobson, E.S. 1974
Chaetodontidae	*Forcipiger flavissimus*		Sulawesi, Indonesia	Personal observation of feeding behavior
Chaetodontidae	*Heniochus varius*	6	Ryukyu Islands, Japan	Sano, M., 1989
Chaetodontidae	*Chaetodon vagabundus*		Sulawesi, Indonesia	Personal observation of feeding behavior
Pomacanthidae	*Centropyge vroliki*	40	Papua New Guinea	Eagle, J.V., & Jones, G. P., 2004
Pomacanthidae	*Pomacanthus imperator*	>50	Maldives	Anderson, C. & Hafiz, A., 1987
Pomacanthidae	*Pomacanthus xanthometopon*	>50	Maldives	Anderson, C. & Hafiz, A., 1987
Pomacanthidae	*Pygoplites diacanthus*	>50	Maldives	Masuda, H. & Allen G.R., 1993
Pomacanthidae	*Centropyge bicolor*		Sulawesi, Indonesia	Personal observation of feeding behavior
Pomacanthidae	*Centropyge tibicen*		Sulawesi, Indonesia	Personal observation of feeding behavior
Pomacentridae	*Chrysiptera rex*		Sulawesi, Indonesia	Personal observation of feeding behavior
Labridae	*Halichoeres porsopeion*		Sulawesi, Indonesia	Personal observation of feeding behavior
Nemipteridae	*Scolopsis bilineata*		Sulawesi, Indonesia	Personal observation of feeding behavior
Zanclidae	*Zanclus cornutus*	84	Hawaii, USA	Hobson, E. S., 1974

## Discussion

Sponges play key functional roles in coral reef ecosystems and identifying the variables that influence their distribution, abundance and diversity patterns is important for our understanding and predictions of the effects of anthropogenic impacts on coral reefs. Earlier studies of sponge distribution and abundance patterns have identified a number of important abiotic and biological variables, however, few studies have examined the effects of multiple variables and their relative importance concurrently. We aimed to determine the variables correlated with the distribution and abundance patterns of sponges on an Indonesian reef system, and to determine the direction of these relationships. Initially, we found that by modeling overall sponge abundance we were unable to explain most of the variation across the study sites. However, further analysis demonstrated that this was most likely due to contrasting responses to sedimentation of the most abundant species in the study, *Lamellodysidea herbacea*, compared to all the other sponge species. In contrast to abundance patterns, sponge assemblage patterns were explained by a larger number of variables with no single dominant driver. Importantly, we also found that sites with the lowest coral cover and highest sedimentation rates were characterized by sponges rather than by any other group of benthic organisms. Although sponge diversity at these sites was lower, overall sponge abundance was just as high as at sites with high levels of coral cover. Given the low coral cover at the high sediment sites, that have previously had much higher levels of coral cover (30–35% in 2002) [66], we propose that some sponge species have the potential dominate sites where environmental quality has declined. It has been proposed that many coral reefs may become sponge reefs in the future in response to ocean warming and acidification [13]. Our study further supports this hypothesis in that further reductions in environmental quality through increases in sedimentation may also favor sponge-dominated reefs; however, they will likely be low diversity systems.

Our study is consistent with earlier studies in tropical systems that have found that sponge abundance and diversity are strongly correlated with sedimentation levels including an earlier study in the Wakatobi [18], [23], [25], [67], [68]. There are a number of potential ways in which sedimentation could affect sponge abundance and diversity. Increasing sedimentation levels in aquaria has been shown to reduce the pumping rate of a tropical sponge [69], which is likely to have negative effects on sponge physiology. There is also evidence that, under conditions of dispersed flow with silt deposition, sponges in experimental aquaria crawled significantly longer distances on the substratum than their control counterparts, most likely to locate microhabitats with lower exposure to silt [70]. Artificially increasing the sediment levels settling on sponges *in situ* can also have a negative effect on sponge growth rates and reproductive status with sponges subjected to increased silt levels containing lower numbers of spermatocytes than those in control treatments [71]. In a field experiment on a Mediterranean sponge species, exposure to sediment significantly reduced the longevity and success of young sponge recruits [72]. However, in contrast to these reports of the negative effects of sedimentation on sponges, a number of studies have reported high levels of sponge species richness and abundance in sedimented environments [24], [25], suggesting many species are tolerant to sedimentation. In addition, sedimentation has also been implicated in mediating competition effects between sponges and other benthic organisms, whereby negative effects of sediment on sponge competitors can enhance the success of sponges [24]. We propose that increased sedimentation rates have the potential to benefit some sponge species and may even have the potential to drive changes to sponge-dominated reef communities, especially when *Lamellodysidea herbacea* is present. When we examined the abundance patterns of the most common sponge in our study, *L. herbacea*, we found that it was positively correlated with sedimentation. This species was present at all the sites we surveyed, but was very abundant at Sampela 1 and Sampela 2, the sites with the lowest levels of coral cover and considered the most degraded. Interestingly, *L. herbacea* was also reported to be the most abundant sponge species at Derawan Islands, also in Indonesia [73]. The reason for the success of this species at highly sedimented sites is unclear and perhaps surprising since it is phototrophic, however, sedimentation rates at Sampela show considerable seasonal variability, with the highest sediments during June-October [48]. Therefore it is possible that this species may be able to tolerate sediment since it does not experience sedimentation impacts all year round. The high abundance of *L. herbacea* at the highly sedimented sites might suggest it has some adaptations to cope with sedimentation, such as the ability to close its ostia [74] or morphological adaptations. It is also possible that *L. herbacea* may have evolved other mechanisms to keep its surface free from sediment, such as mucous production see [75] as this species is rarely observed to have sediment on its surfaces (pers obs ALP, JJB). However, the role of mucous in the prevention of sediment build up on tropical sponges has not been fully explored.

The other important variables that we found associated with sponge abundance were substrate angle and spongivorous fish abundance. In general, we found a positive relationship between surface angle and sponge abundance, which is consistent with an earlier smaller scale study in the Wakatobi [25]. The effect of substrate angle on sponges is most likely to be indirect through its interaction with sedimentation, light availability and the differential effect of these variables on other organisms affecting the rate and potential outcome of spatial competition between these organisms and sponges. Finally, sponge abundance excluding *Lamellodysidea herbacea* was negatively correlated with spongivorous fish abundance. Although there is a growing body of evidence in the Caribbean that fish predation can affect the distribution and abundance of some species [35], [36], little is known about the importance of sponge predation in the Indo-Pacific, although our modeling approach does suggest that it may be important and warrants further examination.

When examining differences in the sponge assemblages at the study sites we found species diversity was lowest at Sampela 1 and Sampela 2. The species rank abundance curves also showed that these sites had fewer numbers of species present and were dominated by one highly abundant species (*Lamellodysidea herbacea*). The species occupancy-abundance plot shows that the species that were the most abundant were also widespread. The most common species *Lamellodysidea herbacea* was present at every site not just the Sampela sites so appears to be a generalist species able to tolerate a wide range of conditions. Our best model explaining differences in sponge assemblage composition among quadrats included sedimentation, chlorophyll-a, fish spongivore abundance, flow and temperature. However, unlike our model of sponge abundance, no single variable explained much more variation than the others and we were able to explain less variation in overall sponge assemblage structure than in the overall patterns of sponge abundance. One of the reasons for this is the exceptionally high sponge diversity in this area [50], [51] that inevitably leads to high variability in sponge assemblages between quadrats. Given that our results show that sponge assemblage variation is related to a number of variables and that each variable explained only a small amount of variation in sponge assemblages, it may be difficult to predict how specific anthropogenic changes will affect overall sponge assemblage structure. Other variables that might account for the unexplained variation in sponge assemblage structure include light levels, wave exposure, competition, and nutrient and food availability (although we did measure Chl-a as a proxy for food availability) as these have all been associated with changes in sponge abundance in other studies [23], [24], [76].

A limitation of this study is the fact that data were collected over a relatively short time period. Biological assemblages are influenced by long-term variation in the local environment and thus our surveys, which were carried out in 2010, only provide a ‘snapshot’ of the predictor variables measured. There is some evidence from other studies in the WMNP carried out over the last ten years that the environmental characteristics that we measured were representative of the spatial variation between the sites. For example, sedimentation rates measured in 2002 were approximately 4 times higher at Sampela 1 than at Kaledupa and 3 times higher than at Buoy 3 [77], which is similar to our findings (sedimentation at Sampela was 3 times higher than Kaledupa and 1.5 times higher than at Buoy 3). Furthermore, monitoring data collected from six of the sites included in this study (S1, K, KDS, B3, PK, R1) by the Indonesian Institute of Marine Sciences (LIPI) and Operation Wallacea show that in 2007 mean hard coral cover was lowest at Kaledupa, followed by Sampela 1, with higher coral cover at the other sites [46]. Therefore, while further sampling of the explanatory variables would be advantageous, particularly as they are likely to vary temporally, we are confident that the data we have collected identifies the relevant spatial variation between sites.

Overall, we found that the degraded sites at Sampela supported as many sponges as the higher quality reefs, although this assemblage had low evenness and was effectively dominated by a single sponge species. Interestingly, despite a major decline in coral cover from 35–40% to currently less than 10% over the last decade, and large declines in herbivorous fish abundance [78], there does not appear to have been any phase shift to an algal-dominated system, which might have been predicted [11]. It is also interesting to note that, sponge densities at Sampela recorded in 2004 were lower (between 60–80 sponges m^−2^), than the present study, suggesting potential increases in sponge abundance over the last decade as coral cover has declined [25]. The fact that the Sampela sites have seen major declines in coral abundance (from >30% to 8–11%) [66] and that sponge abundance appears to have increased provides evidence that the system maybe moving to a more sponge-dominated state. Alternatively, sponges may have always been abundant at these sites and persisted as corals have declined in response to environmental degradation, although this explanation seems unlikely given overall sponge abundance was similar at degraded and high quality reefs, yet diversity was much reduced at the degraded site and dominated by one, apparently sediment tolerant species. Based on our study, and from increasing evidence in the literature e.g. [18], [19], sponge-dominated systems should be considered as a credible future trajectory for some coral reefs [13], and particularly those experiencing heavy sedimentation.

## Supporting Information

Figure S1 **Species accumulation curve.** Species accumulation curve showing the expected mean number of observed species (plus 95% CI) for sample sizes ranging from 1–15 quadrats. For each sample size the means and 95% CI of the species count are obtained from the species observed in 10000 random selections of that samples size from the original 15 quadrats. The dashed line shows the total number of species across the 15 quadrats (n = 58) and the dotted line illustrates the species count for six quadrats.
(TIF)

Figure S2 **Species abundance curves.** Species abundance curves for each study site showing the abundances of all the species observed at each study site ranked from highest to lowest abundance.
(TIF)
