# Supplementary figures and images for: Reduced Diversity and High Sponge Abundance on a Sedimented Indo-Pacific Reef System: Implications for Future Changes in Environmental Quality
Source: PLoS One. 2014 Jan 24;9(1):e85253. doi: 10.1371/journal.pone.0085253 (PMC3901660; doi:10.1371/journal.pone.0085253)

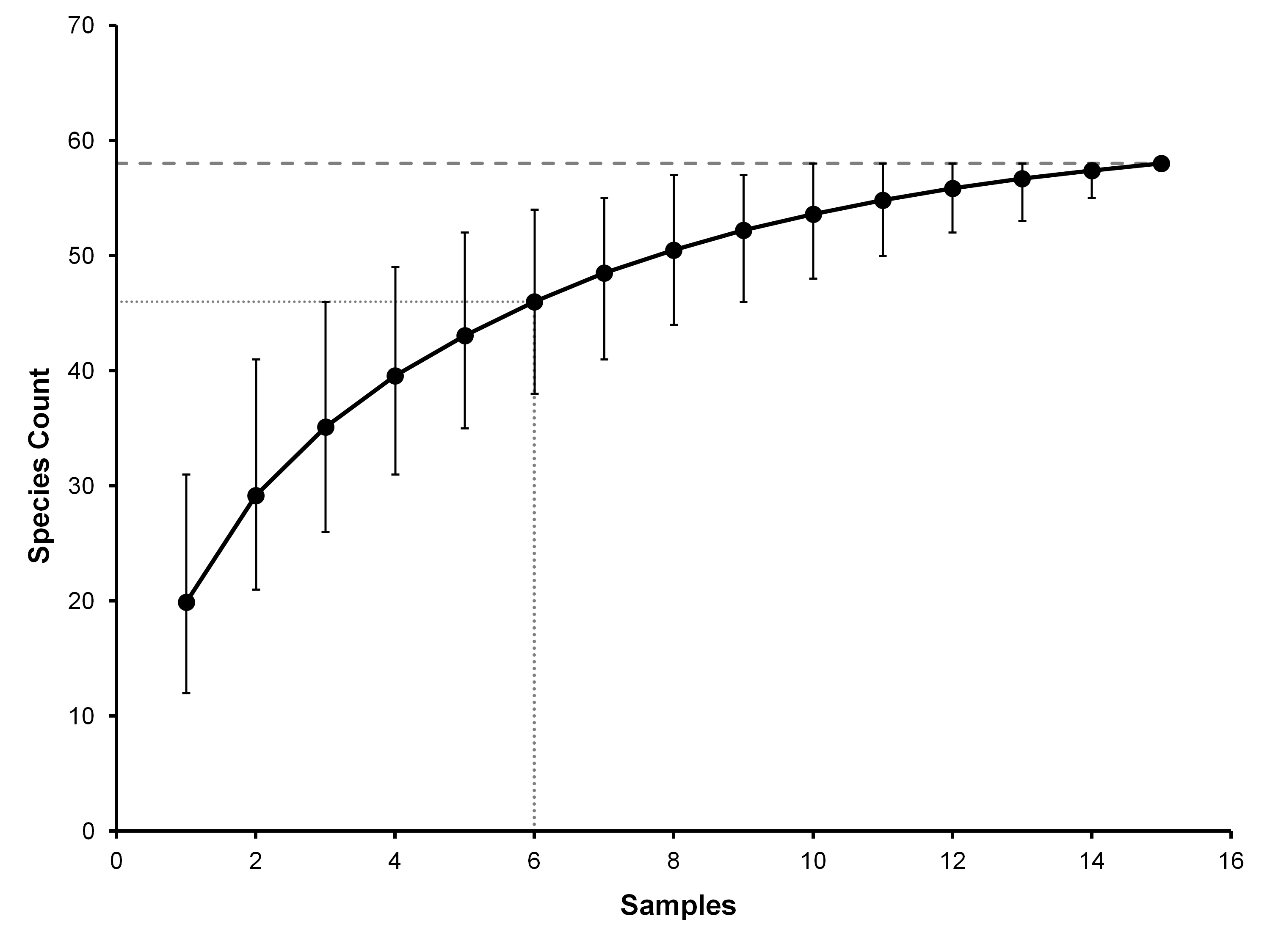

Supplement: Figure S1 — Species accumulation curve. Species accumulation curve showing the expected mean number of observed species (plus 95% CI) for sample sizes ranging from 1–15 quadrats. For each sample size the means and 95% CI of the species count are obtained from the species observed in 10000 random selections of that samples size from the original 15 quadrats. The dashed line shows the total number of species across the 15 quadrats (n = 58) and the dotted line illustrates the species count for six quadrats. (TIF) [file pone.0085253.s001.tif]

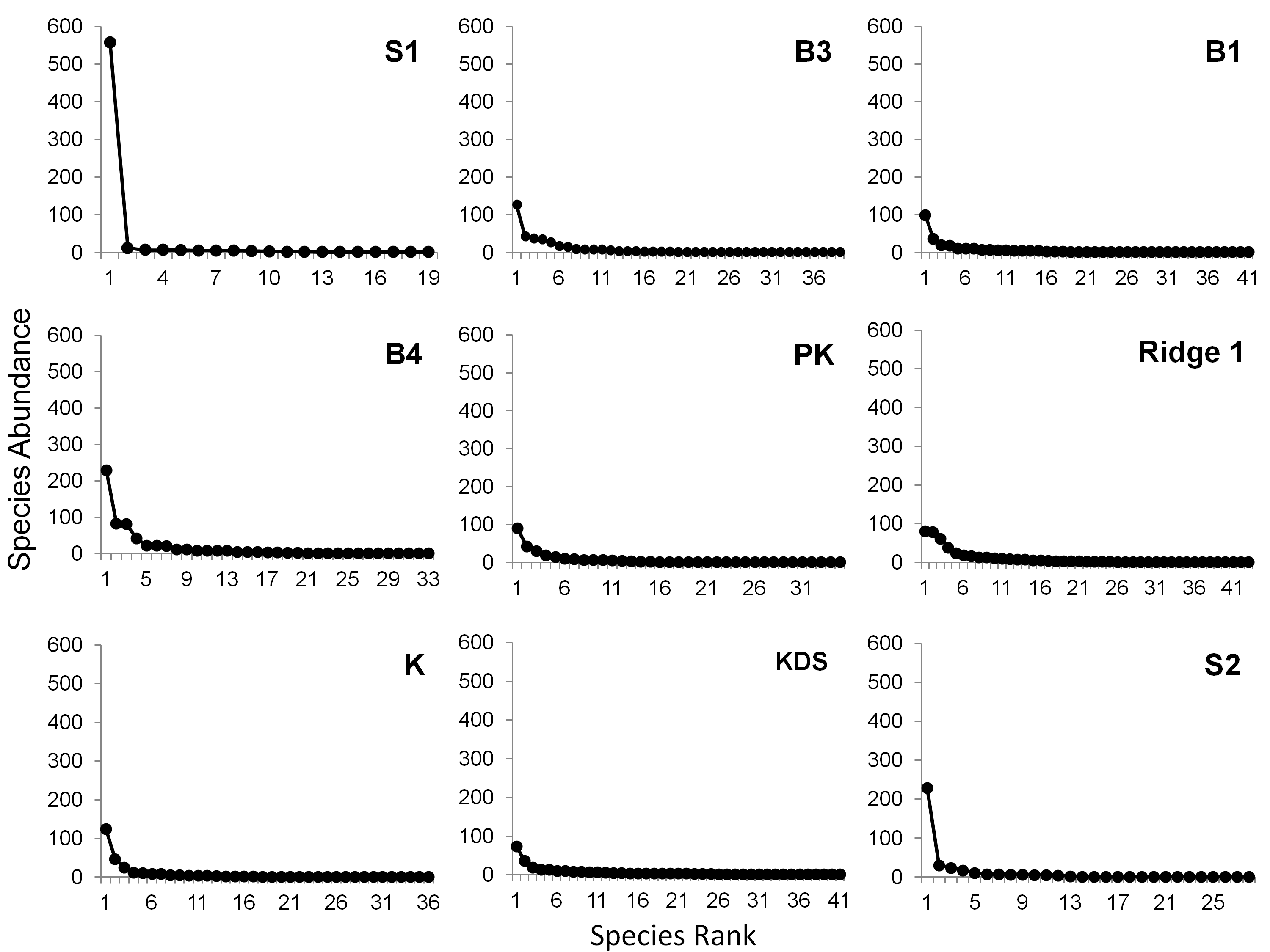

Supplement: Figure S2 — Species abundance curves. Species abundance curves for each study site showing the abundances of all the species observed at each study site ranked from highest to lowest abundance. (TIF) [file pone.0085253.s002.tif]
